# Twice Exceptional in Medicine: A Case Series of Neurodivergent Physicians

**DOI:** 10.1155/crps/8705513

**Published:** 2026-05-21

**Authors:** Ashley Toussaint, Om Panda, Cheryl Graber

**Affiliations:** ^1^ Department of Surgery, Rutgers Robert Wood Johnson Medical School, New Brunswick, New Jersey, USA, rutgers.edu; ^2^ Department of Psychiatry, Rutgers Robert Wood Johnson Medical School, New Brunswick, New Jersey, USA, rutgers.edu

## Abstract

The term twice‐exceptional (2e) describes individuals who combine high intellectual potential with neurodevelopmental or psychiatric conditions that may impair performance in traditional environments. While this concept has been increasingly recognized in education, its relevance to medical training and practice has received little attention. This case series presents five physicians across different stages of training who exemplify twice‐exceptionality, highlighting both their cognitive strengths and the barriers they faced in clinical environments. The first case involves a surgical resident with nonverbal learning disorder (NVLD), attention deficit hyperactivity disorder (ADHD), and type 1 diabetes whose superior intellect masked significant executive dysfunction until repeated crises prompted disclosure and pursuit of accommodations. The second case describes a resident with ADHD and perfectionism who, through structured coping strategies and mentorship, thrived in a supportive environment. A third case highlights a senior resident with anxiety and obsessive–compulsive disorder whose perfectionism and rumination undermined efficiency and self‐confidence despite positive evaluations. The fourth case involves an attending physician with ADHD and obsessive traits who maintained professional success through compensatory overwork but experienced disorganization, fatigue, and burnout. The fifth case describes another attending physician with ADHD, anxiety, and depression whose intellectual giftedness coexisted with executive dysfunction and impaired self‐care, leading to chronic struggles despite ongoing treatment. Across these cases, recurring themes emerge: masking of disability by high achievement, misattribution of neurodivergent struggles as professionalism deficits, and delayed or absent accommodations. While some physicians succeed in supportive environments, others experience isolation, burnout, or attrition when their needs remain unrecognized. These cases underscore the paradox of twice‐exceptionality in medicine, where extraordinary potential and hidden vulnerabilities coexist. Greater recognition of this duality is essential to building neurodiversity‐affirming environments that move beyond deficit‐based models, reduce stigma, and foster structures in which talented but vulnerable physicians can thrive.

## 1. Introduction

The term “twice‐exceptional” (2e) refers to individuals who exhibit both high intellectual potential and one or more neurodevelopmental or psychiatric conditions that may impair performance in traditional learning or professional environments [1]. This duality of exceptional ability and substantial challenge can go unrecognized in medicine, where competence is often narrowly defined, time is compressed, and vulnerability may be stigmatized. While increasingly recognized in other educational contexts, the implications of twice‐exceptionality in high‐stakes environments like medicine remain underexplored and poorly defined in the medical literature [1].

Physician training and practice systems are often ill‐equipped to support neurodivergent learners who struggle not with intellect, but with executive function, anxiety regulation, or social cognition [2]. These challenges are frequently masked by high achievement, leading to underdiagnosis, inadequate support, and unnecessary hardship [3]. This case series presents five physicians at various stages of training and attending practice at a large academic institution who exemplify twice‐exceptionality: each is cognitively capable, professionally motivated, and committed to medicine, yet each has faced significant barriers to thriving in traditional residency environments. These cases highlight the diversity of needs, the consequences of insufficient accommodation, and the possibilities for success with targeted support.

## 2. Case 1: 29‐Year‐Old Male Resident With Nonverbal Learning Disorder (NVLD), Attention Deficit Hyperactivity Disorder (ADHD), and Twice‐Exceptionality

This surgical resident was diagnosed with a NVLD in middle school after neuropsychological testing revealed discrepancies between verbal and performance IQ, with impairment in visual–spatial reasoning and executive functioning. Despite this, he demonstrated a very superior IQ, allowing him to excel academically with accommodations such as extended time on exams. He completed college, an MBA, and medical school with honors (GPA 3.8) and was president of a fraternity—demonstrating strong leadership, resilience, and motivation.

Upon entering residency, however, he began to experience difficulty with the fast pace and unstructured nature of clinical training. He struggled with patient presentations, real‐time clinical decision‐making, and keeping up with documentation. Fellow residents became frustrated with his inefficiencies, particularly when his delayed note completion extended the workday for others. The social dynamics deteriorated, and he became increasingly isolated. He described freezing during feedback sessions, unable to articulate responses. Despite his strong academic foundation, his confidence collapsed under the strain of unrelenting criticism, interpersonal rejection, and lack of support.

He eventually disclosed his history to the program director, but only after reaching a critical point: multiple warning meetings, formal notices of possible dismissal, and worsening mental health symptoms, including anxiety, depression, suicidal ideation, and paranoid thoughts. At no point during training had he been offered disability accommodations or workplace adjustments. He had been undertreating his ADHD and managing labile blood sugars (type 1 DM) without routine breaks due to a lack of time.

Through the Office of Employment Equity, he began pursuing formal accommodations and took medical leave. He engaged in executive function coaching and ADHD medication optimization and began psychotherapy focused on anxiety management. He reported thriving during solo call days—when he could focus without judgment or comparison. He is now considering repeating the intern year with accommodations in place and a more supportive environment and remains committed to continuing in medicine.

## 3. Case 2: 29‐Year‐Old Male Resident With ADHD, Perfectionism, and Twice‐Exceptionality

Diagnosed with ADHD during medical school, this resident had developed a robust repertoire of coping strategies prior to starting residency. He maintained consistent treatment with a psychiatrist and was open about his diagnosis with trusted mentors. His symptoms include distractibility, time blindness, and difficulty shifting tasks. He manages these with a highly structured routine, checklist systems, and scheduled breaks.

Despite his ADHD, he reports that he loves going to work and finds meaning in the clinical environment. His program has been supportive, and his colleagues have helped him adjust during the intern year. While he initially had some challenges with task completion and workflow efficiency, these were addressed through informal feedback, and he actively implemented strategies to improve. Senior residents commented on his progress over the year, especially his willingness to ask for help and delegate when needed.

He continues to work on responding to communication in a timely manner, resisting perfectionism in his routines, and accurately estimating how long tasks will take. However, none of these issues have escalated to formal concern. He has not required formal accommodations and has sustained functional performance and strong evaluations. His case illustrates how self‐awareness, environmental fit, and mentorship can create an affirming training experience for a 2e learner.

## 4. Case 3: 35‐Year‐Old Male Resident With Anxiety, OCD, Obsessional Thinking, and Twice‐Exceptionality

This senior resident presented with a complex psychiatric history, including anxiety, major depressive disorder, and obsessive–compulsive disorder with intrusive thoughts. He has struggled with ruminative self‐doubt, perfectionism, and avoidance behaviors that delay task completion and reinforce feelings of inadequacy.

Despite no negative evaluations, he lives in persistent fear that he is a poor doctor. He describes being “paralyzed” by the fear of missing a detail, walking into a situation unprepared, or being exposed as incompetent. These thought patterns contribute to avoidance and inefficiency, especially during periods of independent work. He delayed his transition to research due to embarrassment over a poor in‐service exam performance, which he experienced as personal failure rather than a momentary lapse.

He reports guilt, shame, and emotional storms, sometimes describing a desire to flee or disappear rather than face responsibilities. Although he seeks high‐intensity clinical roles, his underlying anxiety often blocks his ability to engage fully. Therapy has focused on cognitive restructuring of perfectionistic expectations, reducing “what‐if” thinking, and managing compulsive avoidance. While high‐functioning, he acknowledges that his anxiety impedes his ability to feel like the resident—and physician—he knows he could be.

## 5. Case 4: 38‐Year‐Old Male Attending Physician With ADHD, OCD Traits, and Twice‐Exceptionality

Now a practicing physician, this individual has a long‐standing history of inattention, distractibility, and overthinking, recently conceptualized as adult ADHD with obsessive traits. He has a medical history of type 1 diabetes and Kawasaki disease and is otherwise physically healthy.

Although his professional productivity has remained high, he experiences significant internal disorganization and executive overload. He reports losing objects (e.g., car keys, hospital ID, and parking passes), becoming distracted by minor tasks, and taking excessive time to write emails or respond to messages—often crafting paragraph‐long responses to simple questions. He describes his brain as “exhausted,” especially at home, where decision fatigue leads to friction with his spouse.

He has no overt compulsions but experiences ruminative perfectionism and frustration with his inefficiency. He compensates through long work hours, mental effort, and overpreparation. Despite being respected by colleagues, he feels mentally taxed by the demands of clinical practice and is considering pursuing formal assessment for executive function coaching.

His case highlights the long‐term sustainability challenges for 2e physicians who have adapted without support. Over time, compensatory strategies may erode, leading to burnout or interpersonal difficulties outside of work.

## 6. Case 5: 33‐Year‐Old Male Attending Physician With ADHD, Generalized Anxiety, and Twice‐Exceptionality

This attending physician, now several years into independent clinical practice, has a long‐standing history of ADHD, generalized anxiety disorder, and recurrent major depressive disorder. Throughout his education and early career, he demonstrated hallmarks of twice‐exceptionality—possessing intellectual giftedness alongside significant neurodevelopmental and psychiatric vulnerabilities. Neuropsychological and clinical assessments have suggested neurodiversity, with intact abstract reasoning, strong verbal insight, and a high level of professional motivation.

Despite these strengths, he has consistently struggled with executive dysfunction in high‐pressure environments. His clinical responsibilities often trigger patterns of avoidance, procrastination, and task paralysis—especially when dealing with uncertainty or unstructured demands. These difficulties are compounded by obsessive–compulsive tendencies and pathological demand avoidance, which exacerbate cycles of stress, guilt, and perfectionism.

He has reported persistent day–night reversal, difficulty initiating sleep, and intrusive worries related to sociopolitical events and interpersonal stressors. His capacity for focused, high‐quality work remains evident, particularly during times of autonomy and clarity. However, his challenges with time management, planning, and emotional regulation have impaired his ability to function efficiently in a demanding clinical environment.

In addition to psychiatric diagnoses, he manages type 1 diabetes with the aid of an insulin pump and continuous glucose monitor. He has acknowledged that the rigors of clinical work often interfere with consistent self‐management, including missed meals and erratic glucose control. Pharmacologic treatment includes a combination of stimulants, antidepressants, anxiolytics, and sleep aids (e.g., Concerta, atomoxetine, fluoxetine, bupropion, buspirone, and trazodone).

Despite these challenges, he is highly introspective and engaged in ongoing psychiatric care. He has responded positively to supportive therapy that emphasizes executive function coaching, structured medication schedules, and insight‐oriented work. He reports increased productivity and emotional resilience when external demands are minimized and structure is provided.

This case highlights the long‐term struggles faced by 2e physicians whose cognitive abilities and determination often mask internal disorganization and psychiatric distress. Without workplace accommodations, these individuals are at increased risk for burnout, medical errors, and disengagement. With targeted support and systems‐level awareness, they can continue to thrive in practice while maintaining their well‐being and clinical excellence.

## 7. Discussion

This case series provides a rare window into the experiences of 2e physicians, revealing the profound disconnect between their potential and the structural realities of medical training. The core paradox of 2e involves the co‐occurrence of exceptional intellectual abilities with neurodevelopmental challenges [4], a duality that creates significant hurdles in environments not designed for neurodiversity [5]. A primary theme emerging from these cases is the “masking effect,” where high achievement conceals underlying disabilities [3]. This was particularly evident in Case 1 and Case 3, whose strong academic records and intellectual capacity delayed the recognition of their struggles. The literature confirms that such masking is common, making early and accurate identification exceptionally difficult without sensitive screening methods that look beyond surface‐level performance [6]. The developmental trajectory of 2e individuals is often asynchronous [7], a factor that standard assessments may fail to capture. Furthermore, educators often lack the training to recognize complex 2e profiles, holding beliefs about giftedness that can prevent them from seeing coexisting disabilities [8].

The consequence of this diagnostic ambiguity is that neurodivergent behaviors are frequently misinterpreted. The struggles of the resident in Case 1 were viewed as professionalism deficits, a common and damaging misattribution within medical education that can lead to negative evaluation cycles and immense psychological distress [9]. This problem is compounded by the lack of a formal, universally accepted definition for twice‐exceptionality in major diagnostic manuals like the DSM or ICD [1]. This “diagnostic limbo” creates significant systemic barriers, as access to accommodations and legal protections is often contingent on a formal diagnosis [4]. The significant phenotypic overlap between conditions such as ADHD, autism, and OCD further complicates the diagnostic picture [10–12]. This complexity has led to calls for a more nuanced, transdiagnostic, and dimensional approach to neurodevelopment [13, 14], which would better capture the spectrum of strengths and challenges inherent in 2e individuals. Such a framework would align with neurobiological findings suggesting that the interplay of genetics and neuroplasticity can give rise to both exceptional abilities and functional impairments simultaneously [2].

In the absence of systemic understanding, the burden of navigating medical training falls heavily on the individual. The resident in Case 1 hesitated to disclose his diagnosis until reaching a crisis point, an experience that reflects a well‐documented fear of stigma among resident physicians [15]. Research shows that residency programs often have inadequate disability policies and lack faculty trained to support neurodivergent learners, creating an environment where disclosure feels unsafe [16, 17]. This institutional failure places undue pressure on trainees and can lead to the attrition of uniquely talented individuals [18]. In contrast, the success of the resident in Case 2 demonstrates the power of a supportive ecosystem. His outcome was scaffolded by proactive personal agency and self‐advocacy [19], skills that are critical for 2e individuals. However, his success was not achieved in isolation; it was crucially supported by a receptive environment that included strong mentorship. The literature is unequivocal on the transformative role of aligned, inclusive, and culturally humble mentorship, which provides not only guidance but also the validation and psychological safety necessary for professional identity formation [20–22].

The long‐term consequences of an unsupported journey are evident in Case 4 and Case 5, who experience burnout despite established careers. Their stories highlight the erosion of compensatory strategies and the immense psychological burden of constantly adapting to an unaccommodating world [23, 24]. This internal toll is a recurring theme in the literature, which emphasizes the need for support systems that foster resilience [4]. Fortunately, a growing body of evidence points toward effective interventions. Practical tools like customized note‐taking, scripting, and other methods of externalizing cognition can significantly reduce cognitive load [25, 26]. While generic cognitive training has shown limited generalizability [27, 28], individualized, strategy‐based coaching is highly effective for neurodiverse populations [29]. The positive impact of these formal accommodations on performance and well‐being is well‐supported [30].

Ultimately, these cases call for a paradigm shift in medical education, moving from a deficit‐based model toward a neurodiversity‐affirming framework that values cognitive difference as a source of strength [31, 32]. Recognizing the unique creative and problem‐solving abilities associated with conditions like ADHD and autism is the first step [33]. The goal should not be mere accommodation but the creation of universally designed learning environments that are more just and effective for all [34]. By embracing this perspective, medical institutions can cultivate the full range of human potential, enriching the entire professional community.

## Funding

This research received no external funding.

## Ethics Statement

This case report was conducted in accordance with institutional ethical standards. Written informed consent is not required for a case series based on institutional standards.

## Conflicts of Interest

The authors declare no conflicts of interest.

## Data Availability

Data sharing is not applicable to this article, as no datasets were generated or analyzed during the current study.
